# Sudden unilateral visual field loss

**DOI:** 10.4103/0974-2700.55352

**Published:** 2009

**Authors:** Robin G Jones, Adrian Peall

**Affiliations:** Department of Eye and Rheumatology, Dunedin Public Hospital, Dunedin, NZ; 1 Department of Eye and Rheumatology, University Hospital of Wales, Cardiff, UK

**Keywords:** Branch retinal artery occlusion, embolus, stroke

## Abstract

We report a classical case of branch retinal artery occlusion (BRAO) in the acute setting and review the literature relating to the diagnostic, therapeutic and prognostic facets of this condition. BRAO can cause sudden visual loss and is not an infrequent presentation to emergency medical services. BRAO may indicate predisposing and related conditions capable of significant morbidity and mortality. Although current therapeutic practices in the acute setting are of uncertain benefit, conservative measures may be attempted in the emergency room by a nonophthalmologist with the aim of dislodging the causative embolus. Regardless of the current means of acute management, anitplatelet therapy and cardiovascular risk management remain the mainstay of treatment for BRAO. The potential for life-threatening systemic associations necessities investigation and multidisciplinary input.

## INTRODUCTION

A BRAO occurs when a branch of the central retinal artery becomes occluded, preventing perfusion of an area of the inner layers of the retina. Emboli are the most common cause and subsequent ischemia results in intracellular oedema secondary to cellular injury and necrosis. Presentation is with sudden onset, painless loss of monocular vision. Issues relating to acute presentation and management of BRAO will be reviewed here.

## CASE REPORT

**Figure 1:**
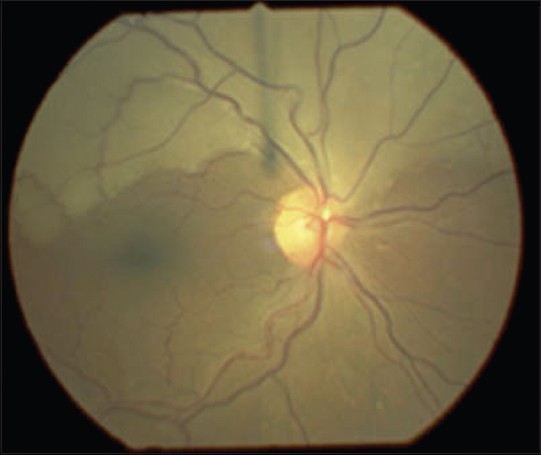
Fundus photograph of right retina

Mr F, a 67-year-old man with a history of coronary and peripheral atherosclerotic disease, presented to the emergency department complaining of a sudden and painless loss of the inferior field of vision in his right eye, initially noted on closure of his left eye 12 h previously. Mr F had an unremarkable medical history, save for having been a smoker for 40 years and having had angina and subsequent coronary angioplasty and placement of stent 6 years previously. His general practitioner had put him on simvastatin, aspirin and quinapril hydrochlordide. Bedside examination confirmed an inferior altitudinal defect of his right eye. There was no relative afferent pupillary defect and Snellen visual acuity measured 6/9 in both eyes. Systems examinations (including full neurological and cardiovascular examinations) were noncontributory and bedside observations fell within normal parameters. Fundoscopy findings are shown in Figure 1. Ocular massage was performed in the emergency department and urgent ophthalmology review was sought. Complete blood count, glucose and serum lipids were within normal limits. The attending ophthalmologist also attempted ocular massage, but without success. No further acute intervention was performed and Mr F returned for echocardiography and Doppler ultrasonography of his carotid arteries 2 days later. Echo findings were unremarkable but Doppler studies revealed a 78% stenosis in his right common carotid artery and, 1 month later, Mr F underwent a right carotid endarterectomy without complication. Routine ophthalmology outpatient follow-up at 4 months revealed a persistent field defect but stable visual acuity. He continued on previous medication and was referred for smoking cessation assistance.

## DISCUSSION

**Figure 2:**
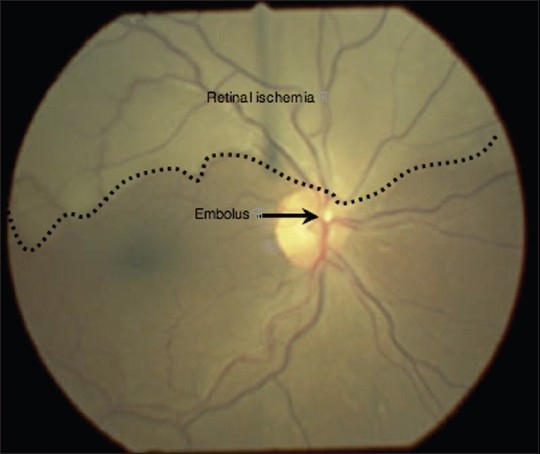
Fundus photograph of right retina with labelled ischemic area and cholesterol embolus (“Hollenhorst plaque”)

Sudden, painless, partial loss of vision in the context of the above clinical signs is suggestive of a branch retinal artery occlusion (BRAO) with macular sparing. The photograph shows a hypopigmented ischemic area of the retina, superior and distal to a large yellow, refractile cholesterol embolus: A “Hollenhorst plaque” [Figure 2]. Retinal veins overlying the affected area remain visible. Obstruction of the retinal arterial circulation is of relevance to both the ophthalmologist and the generalist as visual outcome and ophthalmic complications are associated with systemic disease processes.[1]

Echocardiography and carotid Doppler studies are recommended as emboli frequently arise from atheromatous plaques or thrombi at the carotid bifurcation, internal carotid artery or cardiac valvular structures.[1] Fasting blood glucose and lipids, urine analysis, full blood count, blood cultures and electrocardiography should also be requested. Not all instances of BRAO will present with visible emboli. In such instances, the clinical picture may warrant screening for vasculitis, thrombophilia and syphilis. Giant-cell arteritis does not involve terminal retinal arterioles and is therefore an extremely rare cause of BRAO. Further tests are not indicated in the absence of a suggestive clinical picture.[2]

The goal of treatment in the acute setting is to cause sufficient fluctuation in intraocular pressure to propel an embolus distally along the circulation to a less vital area of supply. Conservative treatment can be attempted in the emergency department and includes intravenous acetazolamide and ocular massage. Anterior chamber paracentesis is frequently performed by ophthalmologists but is of uncertain benefit.[3] More invasive techniques such as ophthalmic artery thrombolysis have been attempted in cases involving the macula, but evidence is limited.[4] It has been suggested that, if initiated within 2-12 h of symptom onset, hyperbaric oxygen therapy may be beneficial.[5] Restoration of retinal blood flow and improved visual function has been reported in patients with BRAO following translumenal Nd:YAG laser embolysis/embolectomy.[6] The recognition of stroke as a complication in patients with retinal emboli means that emphasis is placed on cardiovascular risk management (reduction of serum glucose and cholesterol levels and control of systemic hypertension) and most patients are commenced on antiplatelet therapy, such as aspirin.

## CONCLUSION

Sudden visual loss secondary to retinal arterial occlusion may be the first manifestation of significant atherosclerotic or other systemic disease. Given that persons with retinal emboli are at an increased risk of stroke-related death, a thorough medical and ophthalmological work-up is crucial not only in attainment of diagnosis but also in identification and subsequent treatment of underlying systemic disease. At present, acute management remains an area of controversy as there is no proven treatment for BRAO.
